# Negative Transfer Effects on L2 Word Order Processing

**DOI:** 10.3389/fpsyg.2018.00337

**Published:** 2018-03-14

**Authors:** Kepa Erdocia, Itziar Laka

**Affiliations:** Department of Linguistics and Basque Studies, University of the Basque Country (UPV/EHU), Vitoria-Gasteiz, Spain

**Keywords:** ERPs, word order, second language processing, language distance, syntax, bilingualism

## Abstract

Does first language (L1) word order affect the processing of non-canonical but grammatical syntactic structures in second language (L2) comprehension? In the present study, we test whether L1-Spanish speakers of L2-Basque process subject–verb–object (SVO) and object–verb–subject (OVS) non-canonical word order sentences of Basque in the same way as Basque native speakers. Crucially, while OVS orders are non-canonical in both Spanish and Basque, SVO is non-canonical in Basque but is the canonical word order in Spanish. Our electrophysiological results showed that the characteristics of L1 affect the processing of the L2 even at highly proficient and early-acquired bilingual populations. Specifically, in the non-native group, we observed a left anterior negativity-like component when comparing S and O at sentence initial position and a P600 when comparing those elements at sentence final position. Those results are similar of those reported by Casado et al. (2005) for native speakers of Spanish indicating that L2-Basque speakers rely in their L1-Spanish when processing SVO–OVS word order sentences. Our results favored the competition model (MacWhinney, 1997).

## Introduction

Bilingualism and multilingualism are one of the most important societal challenges for the 21st century. Learning a second language (L2) is a mandatory subject in most countries in the world (UNESCO World Report: Investing in Cultural Diversity Intercultural Dialogue, 2009). All in all, most of the world’s population is capable of communicating in more than one language (Grosjean, 2010). Bilingualism is thus rather common in almost all societies. In this new scenario, research on L2 processing becomes decisive in order to understand how language learning proceeds, and to be able to inform evidence-based language teaching and language policy programs.

One main scientific question in L2 and language learning studies is whether a language learned after the native one is processed through the same neural mechanisms engaged in first language (L1) processing. It is widely accepted that age of acquisition (AoA), proficiency, experience, and typological distance between L1 and L2 may affect L2 processing and its neural representation (for recent reviews see Moreno et al., 2008; Kotz, 2009; van Hell and Tokowicz, 2010; Molinaro et al., 2011; Caffarra et al., 2015). Some linguistic components have shown to be sensitive to AoA like phonology (Flege et al., 1995; Pallier et al., 1997), while other components like semantics have not (Weber-Fox and Neville, 1996; Hahne and Friederici, 2001). In syntax, matters are more contentious: it has been shown that, even at high levels of proficiency, non-natives do not attain native-like competence (Pakulak and Neville, 2010; Díaz et al., 2016). But some studies on syntactic processing argue that at high levels of proficiency, non-native speakers are indistinguishable from native speakers (Birdsong, 1992; Steinhauer et al., 2009) and recruit native-like processing mechanism (Friederici et al., 2002; Mueller et al., 2005; Bastarrika and Davidson, 2017). In this controversy, it is important to determine the role of knowledge of L1 in the processing of L2.

The linguistic distance between L1 and L2, that is, the typological dis-/similarity of the languages involved also plays an important role in the processing of L2 (Kotz, 2009; Zawiszewski et al., 2011). Many studies have shown that L2 speakers can process grammatical traits of L2-like natives do when those traits are identical in L1 and L2, but they cannot achieve native-like processing when those traits are dissimilar from L1 or unique in L2 (Ojima et al., 2005; Tokowicz and MacWhinney, 2005; Foucart and Frenck-Mestre, 2011; Zawiszewski et al., 2011; Erdocia et al., 2014; Díaz et al., 2016). Díaz et al. (2016), for instance, showed that high proficient bilinguals who acquired L2 early in their life (3 years) and low proficient bilinguals who acquired their L2 late (after puberty) display the same electrophysiological responses when confronting in L2 a different argument alignment from their L1 (ergative alignment vs. nominative alignment). That electrophysiological response differed from the one observed in native speakers of Basque (Díaz et al., 2011). However, highly proficient early speakers of Basque displayed the same electrophysiological responses as natives when processing similar syntactic traits (e.g., verb agreement) in both L1 and L2 (see also Zawiszewski et al., 2011). These results are in accordance with the hypothesis that linguistic distance or the degree of typological dissimilarity between L1 and L2 is crucial in L2 learning. *The competition model* for L2 learning of MacWhinney (1997) argues that the entrenchment of L1 may hamper L2 learning. The processing cues shared by L1 and L2 do not compete with each other so the learning of L2 is facilitated by the positive transfer from L1. However, when L1 and L2 differ, their cues compete and learning is difficult, resulting in a negative transfer from L1. Tokowicz and MacWhinney (2005), for instance, observed that L2 learners were sensitive to grammatical violations (e.g., auxiliary omission in progressive tense) of constructions in their L2 when those constructions were formed similarly in their L1 and L2, but they were not sensitive when those constructions were differently formed (see also Sabourin and Stowe, 2008).

The *fundamental difference hypothesis* (Bley-Vroman, 1989) postulates differences in the acquisition of L1 and the learning process of the L2. According to this hypothesis, children acquire L1 implicitly by means of Universal Grammar mechanisms, while L2 learners rely on more general cognitive functions. On the other hand, the *fundamental identity hypothesis* (Hopp, 2010) claims that the grammatical processing of natives and non-natives is similar; and when differences are observed, they must be due to factors related to L1 influence. The *full transfer/full access model* (Schwartz and Sprouse, 1996; White, 2003) proposes that L2 learning initially begins by the transfer of the features available in the L1, and as the bilinguals become more competent in their L2, they progressively gain access to the new features of L2 regardless of their AoA. For instance, Alemán Bañon et al. (2014) showed that L1 English speakers can display P600 effects when processing gender agreement violations in L2 Spanish. Similar results have been reported in studies with German–Italian bilinguals (Rossi et al., 2006), Spanish–English bilinguals (Kotz et al., 2008), and English–French bilinguals (Foucart and Frenck-Mestre, 2012). In a recent MEG study, Bastarrika and Davidson (2017) showed that L1 Spanish speakers can learn syntactic rules of Basque which are not present in Spanish and process them like native speakers of Basque. In that vein, Friederici et al. (2002) showed that a learned artificial language can display native-like biphasic ERP components when processing violations that were not present in the L1 of the participants. For the *dynamic model* (Steinhauer et al., 2009), proficiency rather than AoA seems to predict brain activity patterns in L2 processing, including native-like activity at very high levels of proficiency. Thus, a strict distinction between linguistic structures that late L2 learners can or cannot learn to process in a native-like manner may not be warranted. In that sense, when L2 learners do not reveal native-like processing signatures, they cannot be considered highly proficient (Steinhauer et al., 2009).

The different hypotheses regarding whether bilinguals can process their L2-like natives make different predictions. The competition model and the fundamental difference hypothesis predict an age-related decline in the ability to attain native-like processing of L2, so that the later the exposure to L2 the harder to achieve a native-like attainment. However, these two hypotheses make different predictions regarding the typological distance between L1 and L2. The full transfer/full access model and the dynamic model state that non-native processing can become indistinguishable from native processing. In contrast, the competition model and the fundamental difference hypothesis predict differences when processing dissimilar features between L1 and L2.

### ERP Studies in Bilingual Sentence Processing

Event-related brain potentials (ERPs) have widely demonstrate their validity in L2 processing investigation. The ERP technique provides a direct measure of real-time brain activity at millisecond level. Thus, potentially, ERPs offer the possibility to measure neural activity with a very fine-grained temporal resolution. In that sense, behaviorally non-observable differences between natives and non-natives can be detected by means of this technique, making possible to test the validity of several psycholinguistic models (Moreno et al., 2008).

Event-related brain potentials reflect the activation of large populations of neurons that are activated simultaneously and time-locked to an external event or stimulation (e.g., hearing or reading a word). The ERPs are derived from the electroencephalogram (EEG) that is a continuous variation of oscillations of the electrical activity of the neurons recorded by electrodes placed on the scalp. The information provided by the ERPs is considered multidimensional because it allows the observation of differences in the latency (when the component begins with respect to the stimulation), in the polarity (positive/negative waves), in the amplitude (how positive or negative is the wave), and in the topography (where in the scalp is recorded neuronal activity). Differences in those four dimensions could be taken as reflections of different cognitive processes [for the details on ERP recording and analysis, see Picton et al. (2000) and Luck (2005)]. Three main linguistic components have been proposed considering the mentioned four dimensions and their interpretation in language processing^1^: N400, the left anterior negativity (LAN) and P600.

The N400 is a centroparietal negativity that peaks about 400 ms post-stimulus and it is related to difficulties in semantic processing (Kutas and Hillyard, 1980). Studies on bilingualism showed N400 delays for non-native speakers in comparison to native speakers in semantic violations (Kutas et al., 1994; Weber-Fox and Neville, 1996; Hahne, 2001). Both AoA and proficiency seems to play a role in the delay of the semantic N400 (Moreno et al., 2008).

Left anterior negativity is considered the early syntactic component (Friederici et al., 1993; Weber-Fox and Neville, 1996). Its time window can spread from 150 to 500 ms, and its distribution is left lateralized to the anterior electrodes. LAN is usually associated to automatic and implicit syntactic processing. Phrase structure violations (Weber-Fox and Neville, 1996; Hahne, 2001) and morphosyntactic constraints (Friederici et al., 1993; Coulson et al., 1998) elicit LAN components which have been related to rule-based automatic syntactic processing (Friederici, 2002). Because some variability in the topography has been reported, for instance in bilateral anterior electrodes (Hahne and Jescheniak, 2001; Hagoort et al., 2003) or in left anterior and left central locations (Erdocia et al., 2009), and because the LAN component shares the time window with the N400 component, some LAN effects have been named N400, although they would be closer to a LAN than to a N400 because of the considered syntactic manipulations. In bilingual studies, reductions of the LAN effects (Weber-Fox and Neville, 1996), changes on scalp distribution (Hahne et al., 2006), and absence of effects (Hahne, 2001; Ojima et al., 2005; Hahne et al., 2006; Rossi et al., 2006; Pakulak and Neville, 2010; Liang et al., 2017) have been observed for non-natives in comparison to natives. Those changes or differences between natives and non-natives can diminish as a function of age of first exposure and proficiency (Weber-Fox and Neville, 1996; Steinhauer et al., 2009).

The P600 component has been related to cost of controlled syntactic processing like re-analysis of ungrammatical structures (Osterhout and Holcomb, 1992; Friederici, 2002) and to integration in filler-gap dependencies (Kaan et al., 2000; Phillips et al., 2005)^2^. It is a positive-going wave with a typical central and posterior distribution whose latency begins around 500–600 ms and it can be extended for several hundred milliseconds (van Hell and Tokowicz, 2010). In comparison with the LAN component, the P600 is considered to be more controlled, less automatic (Friederici, 2002). In that sense, studies that tested the processing of L2 syntactic features not present in the bilinguals’ L1 report similar P600 effects for natives and non-natives in contexts where non-natives showed smaller LAN effects than natives [see Caffarra et al. (2015) for an overview]. Some other studies observed a reduction or an absence of the P600 in non-native syntactic processing, suggesting that violations on L2 grammatical structures are not salient enough when those structures cannot been transferred from L1 (Ojima et al., 2005; Chen et al., 2007; Sabourin and Stowe, 2008; Foucart and Frenck-Mestre, 2011; Zawiszewski et al., 2011). Interestingly, studies where different proficiencies of bilinguals are compared showed a change from N400 (for low proficient bilinguals) to P600 (for high proficient bilinguals), revealing the learning process of the L2 (Osterhout et al., 2006; McLaughlin et al., 2010; Foucart and Frenck-Mestre, 2012; White et al., 2012).

### ERP Studies in Bilingual Sentence Word Order Processing

Most ERP studies on L2 processing have used linguistic violation paradigms (Moreno et al., 2008; van Hell and Tokowicz, 2010; Molinaro et al., 2011; Caffarra et al., 2015). Thus, it remains unclear what is the general picture when L2 speakers are confronted with grammatical but unusual (complex) structures in their L2 (Moreno et al., 2008).

Native language studies comparing grammatical sentences with different word order by means of ERPs are scarce. If we focus on sentence word order processing, studies have been carried out on German (Rösler et al., 1998; Matzke et al., 2002; Bornkessel et al., 2002), Spanish (Casado et al., 2005; Nieuwland et al., 2013), Japanese (Hagiwara et al., 2007; Wolff et al., 2008), Chinese (Philipp et al., 2008; Wang et al., 2009), Turkish (Demiral et al., 2008), and Basque (Erdocia et al., 2009). All these studies report that object-before-subject order is harder to process than subject-before-object.

In L2 processing, bilinguals also showed a subject-before-object preference in their non-native language. For instance, Erdocia et al. (2014) tested Spanish native speakers processing Basque subject–object–verb (SOV) and object–subject–verb (OSV) word orders. Comparing the ERPs elicited while processing S vs. O at the initial position of SOV and OSV sentences, non-native participants revealed the native-like processing signature reported in a previous study (Erdocia et al., 2009). However, when comparing O vs. S at the second position of these SOV and OSV sentences, non-natives displayed a P600 component absent in natives. Those results were interpreted to show that non-native processing recruits different neural resources from those used by native speakers, revealing that VO/OV typological distinction between L1 and L2 could be relevant in syntactic computation. Still remains unclear if that difference between natives and non-natives is due to a transfer effect from the native language onto the non-native one (Erdocia et al., 2014).

### The Present Study

In the present study, we investigate how L1-Spanish speakers of L2-Basque process non-canonical SVO and OVS Basque sentences (canonical word order in Basque is SOV; de Rijk, 1969; Erdocia et al., 2009) and how this compares to native processing. Particularly, we seek to find whether L1 Spanish SVO canonical order has an effect in how L2 Basque SVO and OVS non-canonical sentences are processed.

## The Experiment

We investigate word order preferences of native and non-native Basque speakers when processing non-canonical sentence word orders in Basque. If L1 affects the processing of L2, non-native speakers of Basque whose L1 is Spanish should show differences when processing non-canonical SVO and OVS Basque sentences in comparison to Basque native speakers. In that sense, this study aims to investigate whether canonical word order of L1 modulates sentence processing in L2 when the basic word order of L1 and L2 is different.

### Materials and Methods

#### Participants

Two groups of participants were tested: one group of non-native speakers of Basque and one group of native speakers of Basque.

Both groups of speakers signed a written informed consent under experimental protocols approved by the Ethics Committee of the UPV/EHU (Comité de Ética para las Investigaciones relacionadas con Seres Humanos, CEISH), in accordance with the Declaration of Helsinki. All of them were right handed (Edinburgh Handedness inventory: Oldfield, 1971), and they had normal or corrected-to-normal vision. None had previous neurological history.

For the L2 group, 35 L1-Spanish and L2-Basque speakers were recruited for participation in the experiment. All were born in the Basque Country in monolingual Spanish families. They started learning Basque at school (3 ± 1.1 years), and continued their education with Basque as school language. By the age of testing (>18 years), all participants had a C1 level proficiency in Basque^3^. Seven participants were excluded from statistical analysis because of either excessive eye movement or technical problems. Thus, data from 28 highly proficient early learners of L2-Basque have been analyzed (mean age 22 ± 4.6 years; 18 female).

Regarding the native group, 35 native speakers of Basque participated in the experiment. It is worth mentioning that all our adult Basque native speakers were from the southern Basque country located in the northwest of Spain, and thus they all were Basque–Spanish bilingual speakers. According to participants’ self-assessment, they all were born in Basque-speaking families and learned Basque since birth, while they learned Spanish at age of ≈5 years (**Table 1**). Six participants were excluded from statistical analysis because either they had excessive eye movement or because of technical problems. Thus, data from 29 native speakers of Basque have been analyzed (mean age 21 ± 4.9 years; 21 female).

**Table 1:** Comparative between the characteristics of Basque natives’ and non-natives’ sample.

		Self-confidence in							
	AoA (L2)	General		Speaking		Comprehension		Reading	
		Basque	Spanish	Basque	Spanish	Basque	Spanish	Basque	Spanish
Natives	4.9 (±3.3)	6.7 (±0.4)	5.9 (±0.8)	6.7 (±0.4)	5.8 (±.8)	6.8 (±0.4)	6.1(±0.9)	6.6 (±0.5)	6.1 (±0.6)
Non-natives	3 (±1.1)	6.3 (±0.5)	6.6 (±0.4)	6 (±0.9)	6.6 (±0.5)	6.8 (±0.5)	6.7 (±0.4)	6.6 (±0.5)	6.7 (±0.5)

*The age of acquisition (AoA) refers to the L2; in the case of natives to Spanish and in the case of non-natives to Basque.*

Before the experiment, all participants completed a language questionnaire where they reported themselves as very skilled users of Basque. In **Table 2**, we summarize the values reported by the native and non-native speakers taking part in the experiment. We asked participants to evaluate themselves in different linguistic abilities such as speaking, comprehending, or reading, in a 1–7 scale, where 1 was *very bad* and 7 *excellent*. **Table 2** shows that non-native speakers judge their performance in Basque as good as native speakers do.

**Table 2:** Language usage of participants at different periods of their life.

	Use of Basque and Spanish									
	Childhood	First school			Secondary school			University		
	(before school)	School	Home	Other	School	Home	Other	School	Home	Other
Natives	1.3 (±0.5)	1.5 (±0.4)	1.1 (±0.2)	1.7 (±0.7)	1.9 (±0.8)	1.3 (±0.5)	2.3 (±1.1)	2.2 (±0.8)	1.6 (±0.9)	2.9 (±1.1)
Non-natives	6.3 (±1.2)	2.1 (±1.2)	6.1 (±1.2)	5.3 (±1.2)	2.1 (±1.3)	6.3 (±0.7)	5.1 (±1.1)	2.6 (±1.7)	5.8 (±1.2)	4.7 (±1.1)

We also asked participants about their use of Basque at different age periods of their life. From childhood to adulthood, participants reported how much Basque they used at the school, at home, and in other places. Participants addressed their interaction with Basque, from 1 to 7 scale, where 1 was *only in Basque* and 7 was *only in Spanish*.

#### Materials

The experimental materials consisted of 144 transitive sentences containing a subject argument, a direct object argument, and a verb. These sentences were presented in two different non-canonical word order conditions (**Table 3**): SVO condition (1a) and OVS condition (1b). Basque is an ergative language, meaning that subjects of intransitive clauses and objects of transitive clauses are morphologically identical, and bear no overt case ending (called absolutive), while agentive subjects of transitive clauses carry an ergative case marker (-k). In Basque, the transitive verb agrees with the subject and the object, and given that it is a pro-drop language, those elements may be silent (Laka, 1996).

**Table 3:** Example of experimental material.

Experimental conditions	
Subject–verb–object	Object–verb–subject
(1a) Bele-e-k jan dituzte zizare-ak	(1b) Zizare-a jan du bele-a-k
Crow-the(pl.)_ERG._ eat have worm-the(pl.)_ABS._	Worm-the(sg.)_ABS._ eat has crow-the(sg.)_ERG._
“*The crows have eaten the worms*”	“*The crow has eaten the worm*”

Processing our experimental SVO and OVS sentences, participants could rely on morphological information in order to disentangle which word order they were reading. If the first word was marked with the ergative case (-k), participants knew it was the subject. On the other hand, when the first word was not overtly marked (absolutive) they knew it was the object. In order to avoid possible ambiguities [singular ergative and plural absolutive result in the homophonous -ak ending; see Erdocia et al. (2009) for more details], we used plural ergative subjects (-ek) at DP1 position of SVO sentences, and singular absolutive objects (-a) at DP1 of OVS sentences. At DP2 position, we used plural absolutive objects (-ak) in SVO sentences, and singular ergative subjects (-a-k) in OVS sentences; however, DP2s were not ambiguous because the case (ergative or absolutive) of DP1 established the case of DP2. That difference in number forced us to use two different auxiliaries: “dituzte” (plural inflection for S and O) and “du” (singular inflection for S and O).

All in all, we have 144 sentences of SVO word order (1a), 144 sentences of OVS word order (1b), and 288 sentences that act as filler in for the present experiment.

The lexical material of the experimental sentences was controlled for frequency and length using EHME corpus (Euskal Hiztegiaren Maiztasun Egitura/*Frequency Structure of Basque Dictionary*^4^, Acha et al., 2014). The lexical items used as subjects in the experiment generated a value of 0.9 (±0.05) logarithmic frequency and a length of 8.4 letters (±0.18) by average, while the lexical items used as object, 1 (±0.05) for logarithmic frequency, and 8.4 letters (±0.17) for length. The differences between subjects and objects were not statistically significant neither for frequency nor for length (all *P*s > 0.10).

In order to avoid possible overlapping of the electrophysiological effects of the first word in the verb, we included one word postpositional phrase (PP) between those elements, thus example (1a) would be *otsoek mendian jan dituzte ardiak* “lit wolves in-the-mountain eat have the sheep” (see Supplementary Materials).

#### Procedure

Stimuli were divided in four lists. Sentences were randomized (Latin-square) and one version of each item was assigned to one of the four lists (including the fillers). This method allowed every participant to read only once each version of a sentence. The four lists were balanced across participants ensuring that the material was correctly rotated across conditions and participants. This warranted that each version of each sentence was read an equal number of times across participants. Thus, every list had 144 different sentences divided in blocks of eight sentences (18 blocks for list). In every block there were two sentences for each condition plus four fillers. The sentences in each block were mixed randomly every experimental session. Each ERP session lasted about 30–35 min.

Participants were told that the main purpose of the experiment was to read carefully the sentences presented and to resolve correctly a memory task related to the sentences. Experimental trials began with a green asterisk in the middle of the blue screen. The words (in yellow) appeared and disappeared automatically in the middle of the screen until the sentence finished (word duration, 250 ms; stimulus onset asynchrony, 500 ms). Once the sentence finished participants were allowed to blink (during 3000 ms) and the green asterisk appeared again (for 1500 ms) indicating that a new sentence started. Every eight sentences a sentence fragment was presented in brown letters and the participants’ task was to decide whether or not the fragment had been presented in any of the preceding eight sentences. This question remained on the screen until a response was given. This task was used in order to control the attention of the participants during the experiment.

#### ERP Recording

The EEG signal was recorded using a BrainAmp amplifier and the Brain Vision recorder software (Brain Products GmbH, Munich, Germany). The ERPs were recorded from the scalp using tin electrodes mounted in an electrocap (Electro-Cap International) and located at 58 standard positions (Fp1/2, Fpz, F4A/5A, F5/6, F1/2, Fz, C5A/6A, C1A/2A, CZA, C5/6, C1/2, Cz, F3A/4A, F7/8, F3/4, C3A/4A, PZA, T3/4, C3/4, T3L/4L, C3P/4P, P5/6, P1/2, CB1/2B, P1P/2P, Pz, TCP1/2, C1P/2P, T5/6, P3/4, P3P/4P, PZP, O1/2, Oz). EEG data were referenced online to the right mastoid and rereferenced off-line to the mean of the activity at the two mastoid processes. Vertical and horizontal eye movements were monitored with an electrode at the infraorbital and an electrode at the outer canthus of the right eye. Electrode impedances were kept below 5 kΩ.

The electrophysiological signals were filtered with a bandpass of 0.1–30 Hz (half-amplitude cutoffs and digitized at a rate of 250 Hz). The artifact rejection was done automatically by means of the maximum difference of values intervals, where the maximal allowed difference was 150 μV and the interval length was 100 ms. In such cases, 100 ms before and 900 ms after the event were rejected from the analysis. The microvolt range was established between -500 and 500 μV. If the amplitude was higher or lower than such range 100 ms before and 900 ms after the event, this was rejected from the analysis. The baseline was corrected taking into consideration 200 ms previous of the stimulus onset.

#### ERP Data Analysis

For evaluating the scalp distribution of the potential differences between the conditions, 54 electrodes were grouped in nine regions of interest (ROI), six electrodes per ROI: left anterior ROI (LANTE: F3, F3A, F5, F7, C3A, C5A), right anterior ROI (RANTE: F4, F4A, F6, F8, C4A, C6A), left medium ROI (LMD: T3, T3L, TCP1, C3, C3P, C5), right medium ROI (RMD: T4, T4L, TCP2, C4, C4P, C6), left posterior ROI (LPOST: T5, P3, P3P, P5, CB1, 01), right posterior ROI (RPOST:T6, P4, P4P, P6, CB2, 02), middle anterior ROI (MLANT: Fz, F1, F2, CZA, C1A, C2A), middle medium ROI (MLMD: Cz, C1, C2, C1P, C2P, PZA), and middle posterior ROI (MLPOS: Pz, P1, P2, PzP, P1P, P2P).

Stimulus-locked ERPs were averaged for epochs of 1100 ms starting 100 ms prior to the stimulus. First, an omnibus-repeated measures ANOVA was conducted for initial evaluation of the stimulus-locked ERP activity. Lateral and midline ROIs were separate. For lateral ROIs (LANTE, RANTE, LMD, RMD, LPOST, and RPOST), at Hemisphere location (H) two levels were included (left and right), at anterior/posterior location (AP) three levels were included (anterior, medium, and posterior), and for sentence type (ST) two levels were included (SVO and OVS). For midline ROIs (MLANT, MLMD, and MLPOS), Hemisphere factor was removed. When comparing the ERPs of native and non-native speakers, we include the factor GROUP (GR) with two levels: natives and non-natives. Based on the predictions made considering the results of previous experiments in the literature (Erdocia et al., 2009; van Hell and Tokowicz, 2010; Díaz et al., 2016), we chose initially two time-windows of exploration at every sentence’s word positions: 300–500 ms time window for LAN and N400 components, and 600–900 ms time window for the P600 component. Furthermore, we also performed statistical analyses on the average voltage of time windows selected according to visual inspection of the ERP waveforms.

ANOVAs were conducted comparing SVO and OVS sentences. These ANOVAs were carried out for three factors (Hemisphere: two levels, AntPost: three levels, and ST: two levels) in the case of lateral ROIs and two factors (AntPost: three levels and ST: two levels) in the case of midline ROIs. The ANOVAs included the factor GROUP (two levels) for lateral and midline ROIs. For the comparisons, we also paid attention to the visual inspection of the waves; therefore, the time windows chosen for the statistical analysis varied. For each sentence, the first determiner phrase (DP1), the V, and the second determiner phrase (DP2) were analyzed.

For all statistical effects involving two or more degrees of freedom in the numerator, the Huynh-Feldt epsilon was used to correct for possible violations of the sphericity assumption. The exact *P*-value after the correction will be reported below.

## Results

### Behavioral Results

In order to ensure that participants were attentively reading the sentences, they completed a behavioral task while running the ERP experiment. Every eight sentences one other sentence was administered, and participants had to decide whether that last sentences had been shown before in the experimental part. The native group made 3.79 errors out of 18 (*SD* = 1.98), 20.9%. The non-native group made 4.12 errors out of 18 (*SD* = 1.82), 22.92%. The difference between both groups was not significant (*t*(23) = 0.56).

#### Electrophysiological Results

At initial DP1 position (S vs. O for SVO vs. OVS conditions), two marginally significant differences were observed between 300 and 500 ms; one for natives (ST × H × AP *F*(2,56) = 3.37; *P*(HF) = 0.054) and the other for non-natives (ST × H *F*(1,27) = 3.91; *P*(HF) = 0.058). No group effects were observed. Both natives and non-natives showed a larger negative oscillation (LAN-like component) for O of OVS in comparison with S of SVO.

At the second position, the verb, the Basque native speakers displayed marginally significant and significant differences between 400 and 600 ms [lateral locations, ST × AP, *F*(2,56) = 3.93, *P*(HF) = 0.051], 600 and 900 ms [lateral locations, ST × H, *F*(1,28) = 4.15, *P*(HF) = 0.051; ST × AP, *F*(2,56) = 6.71, *P*(HF) = 0.011; midline location, ST × AP, *F*(2,56) = 4.81, *P*(HF) = 0.028], and 700 and 900 ms [lateral locations, ST × H, *F*(1,28) = 4.84, *P*(HF) = 0.036, ST × AP, *F*(2,56) = 9.04, *P*(HF) = 0.003; midline location, ST × AP, *F*(2,56) = 8.16, *P*(HF) = 0.008]. The non-native speakers did not reveal any statistically significant difference. The group analysis revealed that native and non-native speakers differ between 700 and 900 ms time window [lateral locations, ST × AP × GR, *F*(2,110) = 8.21, *P*(HF) = 0.004; midline location, ST × AP × GR, *F*(2,110) = 8.49, *P*(HF) = 0.002]. Thus, native speakers showed a marginally significant delayed N400 component and a P600 component at V position of OVS sentences in comparison to SVO sentences, while non-native speakers did not reveal any ERP effect at that position.

**FIGURE 1:**
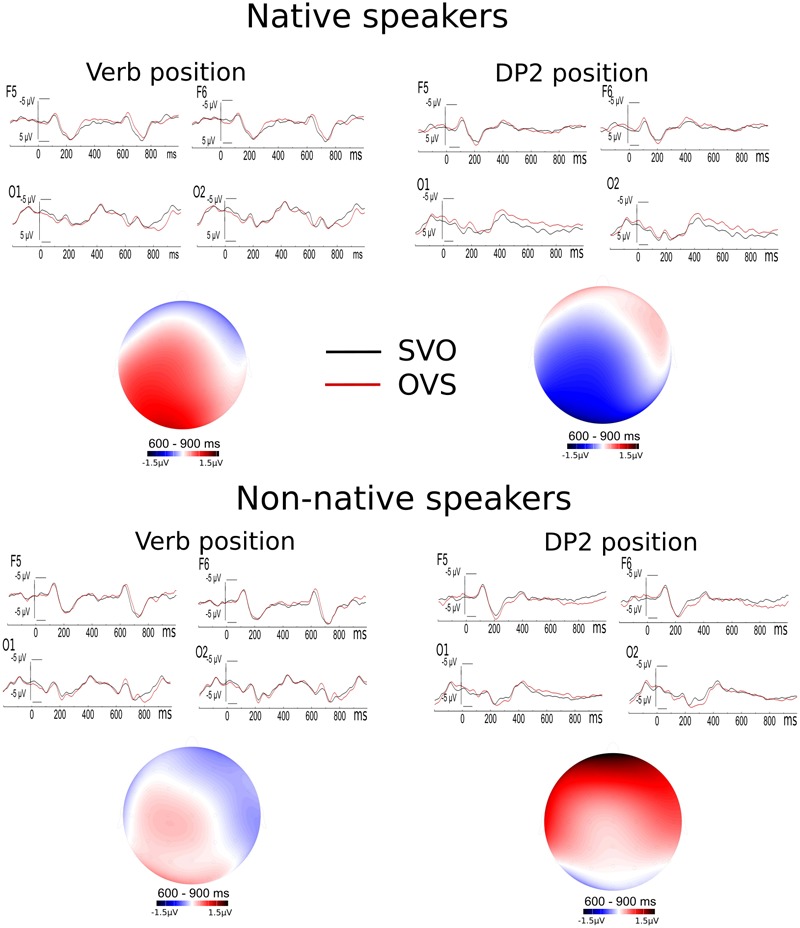
The comparison between SVO and OVS conditions at verb and DP2 positions. Basque native speakers elicit a P600 effect at verb and a long lasting negative effect at second DP position. Non-native speakers only showed a P600 effect at second DP position.

Finally, at the DP2 final sentence position (O vs. S for SVO vs. OVS conditions), statistically significant interactions were observed between 600 and 900 ms for natives [lateral locations, ST × AP, *F*(2,56) = 6.26, *P*(HF) = 0.013; midline locations, ST × AP, *F*(2,56) = 4.70, *P*(HF) = 0.030] and for non-natives [lateral locations, ST × AP, *F*(2,54) = 11.65, *P*(HF) = 0.000; midline locations, ST × AP, *F*(2,54) = 6.02, *P*(HF) = 0.012]. Although natives displayed a negative fluctuation and non-natives displayed a positivity for OVS condition (**Figure 1**), the comparison between those groups of speakers did not reveal any statistically significant difference in that time window. Nevertheless, in the 300–500 ms time window, a ST by group (GR) interaction was observed [lateral locations, ST × GR, *F*(1,55) = 5.39, *P*(HF) = 0.024; midline locations, ST × GR, *F*(1,55) = 4.71, *P*(HF) = 0.034]. The sentence final S generated more negative fluctuations than the O at DP2 position for natives speakers [marginally significant only for the ST factor, lateral locations, *F*(1,27) = *P*(HF) = 0.051], while this effect was not significant for non-natives. Thus, while native speakers of Basque displayed a long lasting N400 at DP2 position of OVS condition in comparison to SVO condition, non-natives displayed a frontal P600.

## Discussion

The results of the ERP experiment revealed electrophysiological differences between native and non-native speakers while processing non-canonical sentences in Basque. On the one hand, native speakers showed a P600 component at V position for OVS condition in comparison with SVO, while non-natives did not show any significant result. On the other hand, at sentence final position (S vs. O at DP2 position), natives showed a long lasting negative component for S (in OVS) in comparison to O (in SVO), while the non-native group presented a P600 for the same comparison. Thus, native speaker showed differences at V and DP2 while non-natives speakers only showed differences at DP2 position, suggesting that non-natives processed easier than natives the non-canonical word orders of Basque. In fact, our results could reflect that knowledge from L1-Spanish is modulating sentence processing in L2-Basque (Schwartz and Sprouse, 1996; White, 2003; Hawkins and Franceschina, 2004; MacWhinney, 2005). That is, the grammar of the L1 grounds the L2 syntactic processing and the neural mechanisms underpinning the L1 have a detectable impact on L2 processing (Caffarra et al., 2015). The influence of L1 over L2 that we report here comes from the differences in canonical word order of the two languages. The canonical word order of Basque is SOV while the canonical word order of Spanish is SVO. The two word orders used in the experiment (SVO and OVS) are not canonical in Basque; however, one of those (SVO) is canonical in Spanish, the L1 of the non-native group. In Spanish, the OVS word order occurs in marked situations when the object is topicalized, though it is perfectly grammatical. In Basque, SVO word order is very common, while the use of OVS word order is less frequent (de Rijk, 1969).

Both groups showed marginally significant LAN-like components at DP1 position for O in comparison with S. Natives and non-natives displayed similar LAN-like components for objects in comparison to subjects at sentence first position. These LAN effects replicate those previously reported in German, Japanese, and Basque for monolinguals when comparing O and S at sentence initial position (Matzke et al., 2002; Hagiwara et al., 2007; Erdocia et al., 2009). The lack of difference between groups at DP1 position when comparing OVS and SVO sentences could be due to the fact that our L2-Basque and L1-Basque speakers applied the same/universal agent-first processing strategy (Bever, 1970; Bornkessel-Schlesewsky and Schlesewsky, 2009; for a recent discussion see Philipp et al., 2017).

Regarding the processing of the verb, natives displayed a parietal P600 for OVS word order sentences in comparison to SVO word order sentences. Although SVO and OVS word orders are non-canonical in Basque, S-first sentences are preferred and more common than O-first sentences (de Rijk, 1969). Thus, that P600 could be interpreted as a reanalysis of the non-canonical OVS word order sentences (Kaan et al., 2000; Friederici, 2002; Phillips et al., 2005). On the contrary, L1-Spanish speakers did not show any P600 at V position of OVS word order sentences in comparison to SVO sentences of Basque. In L2 processing literature, the absence of P600 has been related to proficiency; as the proficiency increases, the P600 appears (Hahne, 2001; Kotz, 2009; Steinhauer et al., 2009). However, our non-native group was highly proficient in L2, so we discard the lack of proficiency as an explanation of the absence of the P600. On the other hand, some studies revealed modulation (or absence) of the P600 when L1 and L2 differ in a given grammatical feature even at high proficiency level (Chen et al., 2007; Foucart and Frenck-Mestre, 2011; Zawiszewski et al., 2011). Those studies suggest that L2 speakers failed to transfer the grammatical structure from their L1 to their L2. However, L1-Spanish speakers are familiar to word order alternations in their L1; thus, Basque and Spanish are not in conflict here. Non-native speakers could rely on their L1-Spanish grammar when processing OVS sentences. If in Spanish, their L1, reading the V at OVS sentences does not generate a P600 (Casado et al., 2005), then our L2-Basque speakers could negatively transfer the processing of Spanish into Basque. In that situation the transfer is negative because Basque and Spanish differ in their canonical word order (SOV vs. SVO) and because the electrophysiological responses of natives and L2-Basque speakers are different, but that transfer could be considered a facilitation of L2 processing because L1-Spanish speakers did not exhibit the P600 that natives did.

Finally, natives and non-natives showed a difference at DP2 position. Natives showed a late posterior N400 for S in OVS sentences compared to O in SVO ones. As we described in the material section, Basque is an SOV pro-drop language; thus, OV constructions are grammatical. Then, the N400 could reflect that native speakers did not expect any lexical material after the verb, being harder to process an agent than a patient at that position. The difference between the agent and the patient could be due to the fact after processing a SV sequence, native speakers of Basque do already know that the O is topicalized, either because it has been dropped or because it will appear after the verb. However, when processing an OV sequence, Basque natives could interpret it as a SOV (canonical) sequence where the S is silent given the pro-drop properties of the language. Then, the S at DP2 position of OVS sentences required a structural reanalysis from SOV to OVS structures, eliciting the N400 observed for the agent but not for the patient. Similar negative components have been reported for S at non-canonical position when comparing SOV and OSV word orders processed by native speakers (Matzke et al., 2002; Erdocia et al., 2009). In the case of non-native speakers of Basque, our participants displayed an anterior P600 in the same comparison indicating a reanalysis of the non-canonical OVS word order sentences (Kaan et al., 2000; Friederici, 2002; Phillips et al., 2005).

In a previous study, Casado et al. (2005) tested native speakers of Spanish processing canonical SVO and non-canonical OVS word orders. They observed a LAN-like component for non-agent-like O in comparison with the agent-like S at sentence initial position. At V position, they did not observe any electrophysiological effect when there was not any disambiguation process going on. Finally, at DP2 position, they observed a P600 for S when comparing canonical SVO and non-canonical OVS word orders^5^. The similarity between the results of native speakers of Spanish (Casado et al., 2005) and the result of our L1-Spanish non-native speakers of Basque provides evidence supporting that the L1 word order is affecting the processing of the L2 word order, and it suggests that the language learned after the native one is processed through the same neural mechanisms engaged in L1 processing.

## Conclusion

The present study showed that bilinguals processed non-canonical word orders in L2 based on their L1’s grammatical restrictions. It favors the *competition model* for L2 processing (MacWhinney, 1997) and the *failed functional features hypothesis* (Hawkins, 2001). We observed that when L1 and L2 differ, their cues compete, resulting in a negative transfer from L1. The canonical word order of L1-Spanish overcomes when processing SVO and OVS word orders facilitating their processing in L2-Basque. Anyhow, we cannot refute the *dynamic account* (Steinhauer et al., 2009) nor the *full transfer/full access model* (Schwartz and Sprouse, 1996) because one could consider that the highly proficient L2 speakers who participated in the present study they do not really reach the highest (native-like) processing level in the particular linguistic trait of word order variation investigated here. Finally, we are not investigating ungrammatical constructions, but correctly formed grammatical structures. Then, it could be that non-native speakers never process the grammatical structures of L2 in a native-like manner, mostly if they already can do it transferring the cues of their L1 grammar. Many works have been done in how humans detect linguistic errors, but more work is required using grammatically correct material.

## Author Contributions

KE performed the research and analyzed the data. KE and IL discussed and interpreted the findings and wrote the paper.

## Conflict of Interest Statement

The authors declare that the research was conducted in the absence of any commercial or financial relationships that could be construed as a potential conflict of interest.

## Footnotes

1 For discussion about the early left anterior negativity (ELAN) component, see Steinhauer and Drury (2012).

2 For further discussion on “semantic P600” see Bornkessel-Schlesewsky and Schlesewsky (2008).

3 Basque Government’s *Euskararen Gaitasun Agiria* (Basque Proficiency Certificate), equivalent to a C1 level.

4 https://www.ehu.eus/ehg/ehme/

5 OVS materials in Casado et al. (2005) have been considered ungrammatical (Demestre, 2012).
