# Supplementary material for: Negative Transfer Effects on L2 Word Order Processing
Source: Front Psychol. 2018 Mar 14;9:337. doi: 10.3389/fpsyg.2018.00337 (PMC5861198; doi:10.3389/fpsyg.2018.00337)
Supplement: Supplementary file 1 [file Data_Sheet_1.PDF]

APPENDIX

The sentences used in the experiment. We included the SVO version of all experimental sentences. The glosses and the literal translation are provided only for the first example. In the rest of the examples we only provided the English translation of the Basque sentences. In Basque, an ergative language, subjects are marked with Ergative case (*ERG*), while objects are marked with Absolutive case (*ABS*). We also included the OVS version (1') of the first example.

1. Otso-ek gau-ean jan dituzte ardi-ak
   Wolf-the(pl)*ERG* night-at eat have sheep-the(pl)*ABS*
   Lit. The wolves at night eat have the sheep
   The wolves have eaten the sheep at night.

1' Ardi-a gau-ean jan du otso-ak
   Sheep-the(sg)*ABS* night-at eat has wolf-the(sg)*ERG*
   Lit. The sheep at night eat has the wolf
   The wolf has eaten the sheep at night.

2. Beleek zuhaitzean jan dituzte zizareak
   The crows have eaten the worms in the tree
3. Krokodiloek uretan jan dituzte oreinak
   The crocodiles have eaten the deers in the water
4. Hontzek lurrean jan dituzte saguak
   The owls have eaten the mice on the ground
5. Azkonarrek oilategian lapurtu dituzte oiloak
   The weasels have stolen the chickens in the henhouse
6. Azeriek ikuiluan lapurtu dituzte untxiak
   The foxes have stolen the rabbits in the farm
7. Ijitoek mendian hartu dituzte behorrak
   The gypsies have taken the mares in the mountain
8. Gaizkileek Txinan lapurtu dituzte umeak
   The criminals have stolen the children in China
9. Gazteek kaiolan garbitu dituzte untxiak
   The young men have cleaned the rabbits in the cage
10. Erizainek ospitalean pisatu dituzte gizonak
    The nurses have weighed the men in the hospital
11. Arrantzaleek azokan pisatu dituzte legatzak
    The fishermen have weighed the hakes in the market
12. Filmeek bideogelan ikaratu dituzte neskatoak
    The movies have frightened the girls in the video-room
13. Kanpaiek elizan txunditu dituzte bisitariak
    The bells have amazed the visitors in the church
14. Bidaiariek zazpietan zeharkatu dituzte itsasontziak
    The travellers have crossed the ships at seven o'clock
15. Garbitzaileek examinetan zeharkatu dituzte pasiloak
    The cleaners have crossed the corridors during the exam period
16. Nomadek udan zeharkatu dituzte zingirak
    The nomads have crossed the drains in summer
17. Katedralek Gaztelan txunditu dituzte apaizak
    The cathedrals have amazed the priests in Castilla
18. Bidaiariek azaroan erabili dituzte hegazkinak

The travellers used the airplanes in November

19. Izarrek iluntzean gidatu dituzte artzainak
    The stars have guided the shepherds at night
20. Ekaitzek neguan beldurtu dituzte eskiatzaileak
    The storms have scared the skiers in winter
21. Kredituek urteetan estutu dituzte emakumeak
    The loans have worried the women for years
22. Trumoiek gauean ikaratu dituzte behiak
    The thunders have scared the cows at night
23. Arranoek zelaiean harrapatu dituzte erbiak
    The eagles have caught the hares in the meadow
24. Katuek salan harrapatu dituzte saguak
    The cats have caught the mice in the living room
25. Igelek zingiran atrapatu dituzte euliak
    The frogs have captured the flies in the pond
26. Trenek kalean harrapatu dituzte morroiak
    The trains have caught the boys on the street
27. Uholdeek mendian harrapatu dituzte autobusak
    The floods have got the buses on the mountain
28. Irakasleek examinetan suspenditu dituzte ikasleak
    The teachers have failed the students in the exam period
29. Hegazkinek Nafarroan suntsitu dituzte urtegiak
    The airplanes have destroyed the reservoirs in Navarre
30. Elurteek abenduan estali dituzte teilatuak
    The snowfalls have covered the roofs in December
31. Lainoek zazpietan estali dituzte harkaitzak
    The clouds have covered the rocks at seven o'clock
32. Ostoek udazkenean estali dituzte harriak
    The leafs have covered the stones on autumn
33. Kolonizatzaileek etxoletan izan dituzte esklabuak
    The colonists have had the slaves in the shacks
34. Lehoiek zelaiean ehizatu dituzte zebrak
    The lions have hunted the zebras on the field
35. Panterek hondakinetan ehizatu dituzte hontzak
    The panthers have hunted the owls on the residues
36. Galderek zazpietan urduritu dituzte hizlariak
    The questions have got nervous the speakers at seven
37. Opariek gabonetan urduritu dituzte mutikoak
    The presents got nervous the boys at Christmas
38. Arazoek eskolan kezkatu dituzte irakasleak
    The problems have worried the teachers at the school
39. Zigorrek epaitegian kezkatu dituzte abokatuak
    The punishments have worried the lawyers in the court
40. Epaiek udaletxean kezkatu dituzte zinegotziak
    The verdicts have worried the city councilors in the city hall
41. Aipamenek hitzaldian mindu dituzte salatariak
    The allusions have offended the informers in the talk
42. Filmeek aretoan gogaitu dituzte zuzendariak

The movies have annoyed the directors in the auditorium

43. Zaparradek maiatzean gogaitu dituzte oinezkoak
    The downpours annoyed the walkers in May
44. Abestiek igerilekuan lasaitu dituzte izurdeak
    The songs have calmed the dolphins in the swimming pool
45. Gizonek tabernan piztu dituzte poxpoloak
    The men have lighted the matches in the pub
46. Sendagaiek hamarretan indargabetu dituzte birusak
    The medicines have weakened the viruses at ten o'clock
47. Zigarroek ospitalean hil dituzte igeltseroak
    The cigarettes have killed the construction workers in the hospital
48. Bonbek ostiralean hil dituzte herritarrak
    The bombs killed the citizens on Friday
49. Hurakanek itsasoan hil dituzte marinelak
    The hurricanes have killed the sailors in the sea
50. Lurrikarek lehorrean hil dituzte indiarrak
    The earthquakes killed the Indians on land
51. Enborrek plazan nekatu dituzte aizkolariak
    The branches have tired the woodcutters in the square
52. Atxurrek baratzean nekatu dituzte morroiak
    The hoe have tired the servants in the vegetable garden
53. Lanek udan nekatu dituzte langileak
    The works tired the workers in summer
54. Haizeteek lurrera bota dituzte aizkolariak
    The strong winds have thrown the woodcutters to the ground
55. Gutunek goizean ekarri dituzte berriak
    The letters have brought good news in the morning
56. Ezkutuek manifan babestu dituzte poliziak
    The shields have protected the policemen in the demonstration
57. Teilatuek iparraldean babestu dituzte etxeak
    The roofs have protected the houses in the north
58. Olerkiek mezetan hunkitu dituzte idazleak
    The poems have moved the writers during the masses
59. Lurrikarek abuztuan suntsitu dituzte herriak
    The earthquakes destroyed the villages in August
60. Lehorteek astelehenean suntsitu dituzte familiak
    The droughts destroyed the families on Monday
61. Ekaitzek Eskozian suntsitu dituzte basoak
    The storms have destroyed the forests in Scotland
62. Eztandek bostetan esnatu dituzte herritarrak
    The explosions have woken up the villagers at five o'clock
63. Telefonoek zortzietan esnatu dituzte gizonak
    The telephones have woken up the men at eight o'clock
64. Deiek goizean esnatu dituzte bonberoak
    The calls have woken up the firefighters in the morning
65. Leherketek seietan esnatu dituzte erizainak
    The explosions have woken up the nurses at six o'clock
66. Aurreskuek festan hunkitu dituzte neskatoak

The aurreskus [Basque dances of honor] have moved the girls in the festivities

67. Olatuek itsasoan ikaratu dituzte surflariak
    The waves have frightened the surfers in the sea
68. Perituek kalean aztertu dituzte laborategiak
    The experts have analyzed the laboratories in the street
69. Mutikoek goizean apurtu dituzte kotxeak
    The boys have broken the cars in the morning
70. Skinek seietan apurtu dituzte motorrak
    The skinheads have broken the motorbikes at six o'clock
71. Punkiek hiruretan puskatu dituzte kamioiak
    The punks have smashed the trucks at three o'clock
72. Bertsoek plazan mindu dituzte entzuleak
    The improvised verses have offended the listeners (audience) in the square
73. Zakurrek mendian bilatu dituzte usoak
    The dogs have looked for pigeons in the mountain
74. Marinelek uretan bilatu dituzte arrainak
    The sailors have looked for fishes in the water
75. Katuek egongelan puskatu dituzte baloiak
    The cats have broken the balls in the living-room
76. Tximistek iluntzean beldurtu dituzte zezenak
    The lightnings have scared the bulls at twilight
77. Mendiek barruan ezkutatu dituzte kobazuloak
    The mountains have hidden the caves inside
78. Leizeek urrian ezkutatu dituzte kutxak
    The caverns have hidden the boxes in October
79. Idazkariek ekainean gorde dituzte liburuak
    The secretaries stored the books in June
80. Kutxek logelan gorde dituzte eskutitzak
    The boxes have stored the letters in the bedroom
81. Neskameek armairuan tapatu dituzte zapiak
    The maids have covered the dusters in the wardrobe
82. Txalupek iluntzean gurutzatu dituzte errekak
    The boats have crossed the streams at twilight
83. Itsasontziek udan gurutzatu dituzte ibaiak
    The ships have crossed the rivers in summer
84. Medikuek ospitalean gurutzatu dituzte pasiloak
    The doctors have crossed the corridors in the hospital
85. Turistek zortzietan gurutzatu dituzte trenak
    The tourists have crossed the trains at eight o'clock
86. Enpresek udaberrian zapaldu dituzte langileak
    The companies have oppressed the workers in spring
87. Trenek tunelean zapaldu dituzte txakurrak
    The trains have crushed the dogs in the tunnel
88. Klubek eguberritan bota dituzte entrenatzaileak
    The clubs have fired the coaches during Christmas
89. Tximinoek hirian bota dituzte paretak
    The monkeys have demolished the walls in the city
90. Kolonoek itsasontzian erosi dituzte esklaboak

The colonists have bought the slaves in the ship

91. Euriteek udaberrian aldatu dituzte ibaiak
    The rains have changed the rivers in spring
92. Lehorteek uztailan aldatu dituzte paisaiak
    The droughts have changed the landscapes in July
93. Paisaiek udazkenean harritu dituzte turistak
    The landscapes have amazed the tourists in autumn
94. Ikuskizunek parkean harritu dituzte umeak
    The spectacles have amazed the children in the park
95. Ekintzek goizean harritu dituzte etsaiak
    The aggressive actions have surprised the enemies in the morning
96. Loreek iluntzean liluratu dituzte maitaleak
    The flowers have charmed the lovers at twilight
97. Gobernuek neguan kontratatu dituzte kamioilariak
    The governments have hired the truckers in winter
98. Udaletxeek abuztuan kontratatu dituzte garbitzaileak
    The city-halls have hired the cleaners in August
99. Aldundiek udazkenean kontratatu dituzte basozainak
    The Provincial Governments have hired the foresters in autumn
100. Diputazioek apirilean kontratatu dituzte bonberoak
    The provincial councils have hired the firefighters in April
101. Baserritarrek apostuan irabazi dituzte zaldiak
    The farmers have won the horses in the bet
102. Terroristek etxean bahitu dituzte neskatxak
    The terrorists have kidnapped the girls at home
103. Poliziek oihanean miatu dituzte turistak
    The policemen have searched the tourists in the jungle
104. Liburuek urtarrilean bildu dituzte bertsoak
    The books have compiled the verses in January
105. Garbitzaileek logelan bildu dituzte armiarmak
    The cleaners have picked the spiders in the bedroom
106. Ikuskizunek zirkoan bildu dituzte pertsonak
    The spectacles have gathered the people in the circus
107. Sokek espetxean lotu dituzte iheslariak
    The ropes have tied up the fugitives in jail
108. Olatuek itsasoan irentsi dituzte etorkinak
    The waves have swallowed the immigrants in the sea
109. Lehoiek sakanan irentsi dituzte oreinak
    The lions have swallowed the deers in the valley
110. Tigreek oihanean irentsi dituzte behorrak
    The tigers have swallowed the mares in the jungle
111. Marrazoek itsasertzean irentsi dituzte hegaluzeak
    The sharks have swallowed the albacores in the coast
112. Ferratzaileek ikuiluan markatu dituzte zaldiak
    The blacksmiths have marked the horses in the stalls
113. Goxokiek eskolan lasaitu dituzte haurrak
    The candies have calmed the children at the school
114. Nominek igandean lasaitu dituzte beharginak

The payslips calmed the workers on Sunday
115. Musuek irlan lasaitu dituzte maitaleak
The kisses have calmed the lovers on the island
116. Mezek goizetan aspertu dituzte kristauak
The masses have bored the Christians in the mornings
117. Ikasgaiek ikastolan aspertu dituzte irakasleak
The lessons have bored the teachers at the school
118. Esperimentuek laborategian aspertu dituzte partaideak
The experiments have bored the participants in the laboratory
119. Azterketek ikastolan aspertu dituzte ikasleak
The exams have bored the students at the school
120. Teknikariek etxean konpondu dituzte ordenagailuak
The technicians have repaired the computers at home
121. Langileek azkenean hobetu dituzte errepideak
The workers have improved the roads at last
122. Emakumeek enpresan hobetu dituzte baldintzak
The women have improved the conditions in the company
123. Medikuek makinan luzatu dituzte besoak
The doctors have lengthened the arms in the machine
124. Ardoek sotoan mozkortu dituzte atsoak
The wines have drunken the old women at the basemen
125. Patarrek festan mozkortu dituzte gazteak
The liquors have drunken the youngsters at the party
126. Garagardoek kotiloian mozkortu dituzte neskatxak
The beers have drunken the girls at the New Year's Eve party
127. Txanpainek gabonetan mozkortu dituzte izebak
The champagnes have drunken the aunts during Christmas
128. Goseteek Afrikan bizkortu dituzte haurrak
The famines have strengthened the children in Africa
129. Ikerlariek laborategian asmatu dituzte robotak
The researchers have invented the robots in the laboratory
130. Zientzialariek lanean asmatu dituzte ordenagailuak
The scientists have invented the computers at work
131. Kanutoek zubian zorabiatu dituzte ertzainak
The joints have got dizzy the policemen in the bridge
132. Ardoek txoznetan zorabiatu dituzte gaztetxoak
The wines have got dizzy the youngsters in the bars
133. Antzezlanek parkean zorabiatu dituzte emakumeak
The plays have got dizzy the women in the park
134. Bidaiek iparraldean zorabiatu dituzte bidaiariak
The trips have got dizzy the travelers in the north
135. Gizonek etxean garbitu dituzte txoriak
The men have cleaned the birds at home
136. Emakumeek sukaldean garbitu dituzte oiloak
The women have cleaned the chickens in the kitchen
137. Baserritarrek hamarretan garbitu dituzte arkumeak
The farmers have cleaned the lambs at ten o’clock
138. Patxaranek zahartzaroan izorratu dituzte gibelak

The Basque liquors have ruined the livers at old age

139. Tenperaturek Errioxan izorratu dituzte ardoak
The temperatures have ruined the wines in Rioja (wine region)
140. Irinek eguerdian loditu dituzte saltsak
The flours have thickened the sauces at noon
141. Suteek basoan uxatu dituzte piztiak
The fires have frightened off the beasts in the forest
142. Tximistek zuhaitzean uxatu dituzte arranoak
The lightnings have frightened off the eagles in the tree
143. Balek gerran zauritu dituzte soldaduak
The bullets have wounded the soldiers in the war
144. Trumoiek azokan uxatu dituzte agureak
The thunders have frightened off the old men at the market
